# Tumor mutation burden and recurrent tumors in hereditary lung cancer

**DOI:** 10.1002/cam4.2120

**Published:** 2019-04-02

**Authors:** Yi‐Chiung Hsu, Ya‐Hsuan Chang, Gee‐Chen Chang, Bing‐Ching Ho, Shin‐Sheng Yuan, Yu‐Cheng Li, Jhih‐Wun Zeng, Sung‐Liang Yu, Ker‐Chau Li, Pan‐Chyr Yang, Hsuan‐Yu Chen

**Affiliations:** ^1^ Department of Biomedical Sciences and Engineering National Central University Taoyuan Taiwan; ^2^ Department of Clinical Laboratory Sciences and Medical Biotechnology, College of Medicine National Taiwan University Taipei Taiwan; ^3^ Division of Chest Medicine, Department of Internal Medicine Taichung Veterans General Hospital Taichung Taiwan; ^4^ Comprehensive Cancer Center Taichung Veterans General Hospital Taichung Taiwan; ^5^ Institute of Statistical Science Academia Sinica Taipei Taiwan; ^6^ Department of Statistics University of California Los Angeles Los Angeles California; ^7^ College of Medicine National Taiwan University Taipei Taiwan

**Keywords:** hereditary lung cancer, mutation load, nonsmoker, recurrence, whole genome sequencing

## Abstract

Lung cancer is the leading cause of cancer death worldwide and cancer relapse accounts for the majority of cancer mortality. The mechanism is still unknown, especially in hereditary lung cancer without known actionable mutations. To identify genetic alternations involved in hereditary lung cancer and relapse is urgently needed. We collected genetic materials from a unique hereditary lung cancer patient's blood, first cancer tissue (T1), adjacent normal tissue (N1), relapse cancer tissue (T2), and adjacent normal tissue (N2) for whole genome sequencing. We identified specific mutations in T1 and T2, and attributed them to tumorigenesis and recurrence. These tumor specific variants were enriched in antigen presentation pathway. In addition, a lung adenocarcinoma cohort from the TCGA dataset was used to confirm our findings. Patients with high mutation burdens in tumor specific genes had decreased relapse‐free survival (*P* = 0.017, n = 186). Our study may provide important insight for designing immunotherapeutic treatment for hereditary lung cancer.

## 1 INTRODUCTION

Although the majority of lung cancer occurred in patients with the history of smoking, about 25% of the lung cancer cases in the world occur in never smokers.^1^ Lung cancer in never smokers is especially prominent in Asian.^2^, ^3^ Full genomic analysis showed that the genetic variation of lung cancer in never smokers is significant different from smokers.^4^ Oncogenic driver mutations such as *EGFR*and *ALK*were frequently identified in never smoking lung adenocarincoma.^5^

The most effective way to cure lung cancer is early diagnosis for treatment by either surgery or local radiotherapy. However, cancer relapse is still the leading cause of cancer‐related deaths in early stage lung cancer patients after surgery. Many studies showed several genes were associated with cancer relapse but a global view of lung cancer relapse by whole genome analysis is lacking. Next‐generation sequencing analysis has become a common approach of discovering mutations across the whole genome.^4^, ^6^ Previous studies indicated genetic heterogeneity between primary tumors and locally recurrent or metastatic tumors.^7^, ^8^, ^9^ We identified a germlinem mutation in YAP1, a key component of the Hippo pathway, for hereditary lung adenocarcinoma.^10^ However, intratumor heterogeneity of normal‐tumor pairs with primary and relapse tumors in has not been systematically characterized by next‐generation sequencing in hereditary lung cancer. We applied whole genome sequencing to study multiple samples from blood, primary and relapse tumors in one lung adenocarcinoma patient with germline YAP1 R331W missense mutation and family history. We investigated the genetic intratumor heterogeneity and found that the immune‐associated gene somatic variants would predict the relapse‐free survival.

## 2 MATERIALS AND METHODS

### 2.1 Clinical and histopathological data

A 52‐year‐old woman without smoking history was diagnosed with stage IA lung adenocarcinoma over left upper lobe of lung after radical lobectomy initially. Two years later, the patient had disease relapse over left lower lobe of lung and the tumor lesion was removed by wedge resection with metastatic adenocarcinoma from lung proved. About 3 months after the second resection, the patient developed multiple metastases over the contralateral lungs and brain. We collected the patient's blood, first cancer tissue (T1), adjacent normal tissue (N1), relapse cancer tissue (T2), and adjacent normal tissue (N2) for whole genome sequencing. The genomic DNA was extracted from the five samples followed by standard protocol, respectively. The study was approved by the Institutional Review Board of Taichung Veterans General Hospital (IRB no. N05160) and informed consent was signed by patient in person.

### 2.2 Identification of key driver mutations

Five oncogenic drivers, including *EGFR*, *KRAS*, *BRAF*, *HER2,* and *EML4‐ALK*, were tested. *EGFR*, *KRAS*, *BRAF,* and *HER2*mutations were assessed by matrix‐assisted laser desorption ionization‐time of flight mass spectrometry (MALDI‐TOF MS). *EML4‐ALK* translocation was tested by Ventana method.^11^ The YAP1 R331W germline mutation was validated in blood DNA by MALDI‐TOF analysis.

### 2.3 Whole genome sequencing analysis

Next generation sequencing mate‐paired libraries were constructed according to the manufacturer's standard protocol (Life Technologies, Foster City, CA). In brief, genomic DNAs were fragmented into ~3 kb in length followed by end repair, adapter ligation and library amplification and the resulting libraries were then used as the templates for emulsion PCR coupling to beads via an adapter sequence. The amplified beads were then processed to the 3' end modification to allow the beads to covalently attach to the sequencing slide. SOLiD^TM^ sequencing primers were hybridized to the adapter sequence and four fluorescent labeled di‐base probes were used in ligation‐based sequencing. Each nucleotide is sequenced twice in two sequential ligation reactions. Single end 50 bases were sequenced for and the sequencing data were mapped to the human genome reference sequence (hg19) using the SOLiDTM BioScopeTM software pipeline. The raw data were available at: http://ifg.stat.sinica.edu.tw/lungcancer_solid/t1t2n1n2.

### 2.4 Bioinformatic analysis

The standard SOLiD software BioScope (Life Technologies, Foster City, CA) was carried out to analyze sequencing data including image analysis, mapping to human reference genome (UCSC Hg19). Alignment files were base quality score recalibrated and locally realigned around indels with GATK^12^ and marked for duplicates using PICARD tools (picard.sourceforge.net). Consensus genotype calls were generated using SAMtools^13^ and GATK 2.7‐2 annotated using the Annovar package.^14^ The somatic variants were distinguished by filtering the 1000 Genomes phase 1.^15^ We defined the novel SNVs were compared with NCBI dbSNP version 138 (http://www.ncbi.nlm.nih.gov/projects/SNP/) to annotate known SNP information. Visualizing genomic data used the Circos.^16^

### 2.5 Functional enrichment and phylogenetic analysis

Analysis for gene list enrichment was using the software package ingenuity pathway analysis (IPA). The functional analysis tool identified the canonical pathways that were based on the genetic variant list. HLA regions were used to determine the best model by MEGA6 17 was used to construct maximum‐likelihood phylogenetic trees.

### 2.6 Public lung cancer data set

The somatic mutation profile and clinical data of 230 lung adenocarcinoma patients (TCGA)^18^ were downloaded from cBioPortal (www.cbioportal.org).^19^, ^20^

### 2.7 Statistical analysis

Paired *t* test was used to compare somatic mutations in T1 and T2 groups. We calculated the patients' mutation load from the tumor specific mutation genes and classified them into the high‐mutation or the low‐mutation groups with the median of mutation frequency as the threshold value. Kaplan‐Meier survival curves were obtained and compared by log‐rank tests. Statistical significance was defined as a *P* < 0.05. All statistical analyses were performed in the R language environment.

## 3 RESULTS

### 3.1 Whole genome sequencing analysis

**Figure 1:**
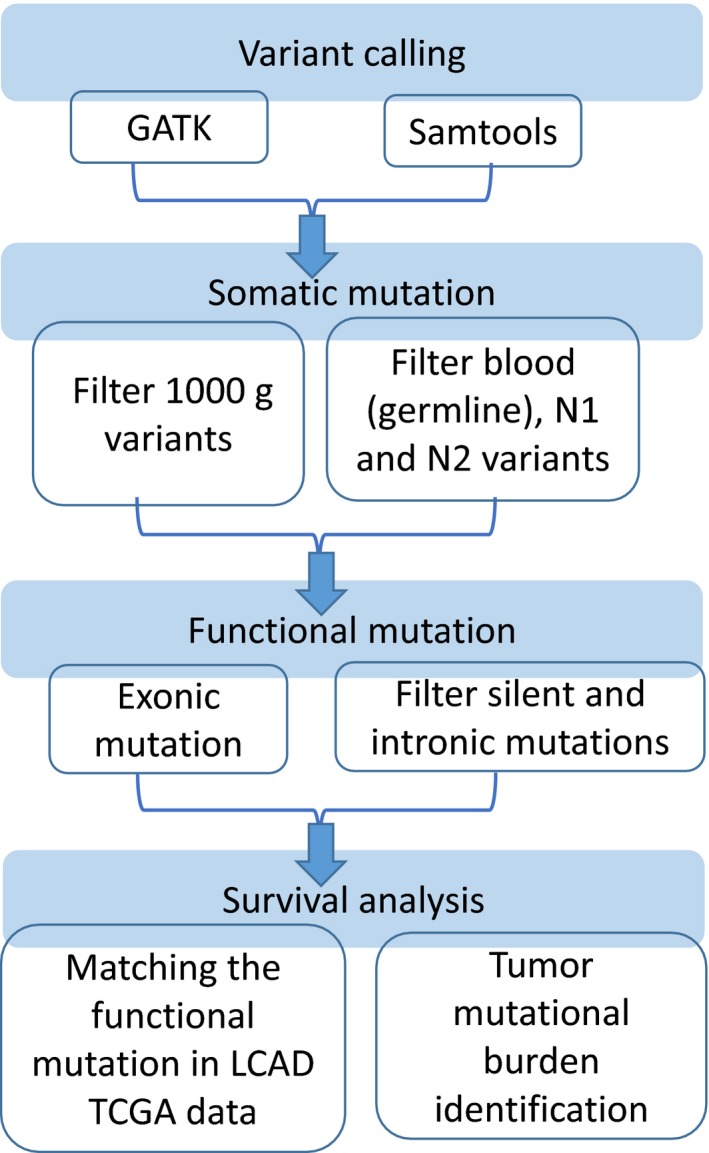
Whole genome sequencing analysis flowchart

**Figure 2:**
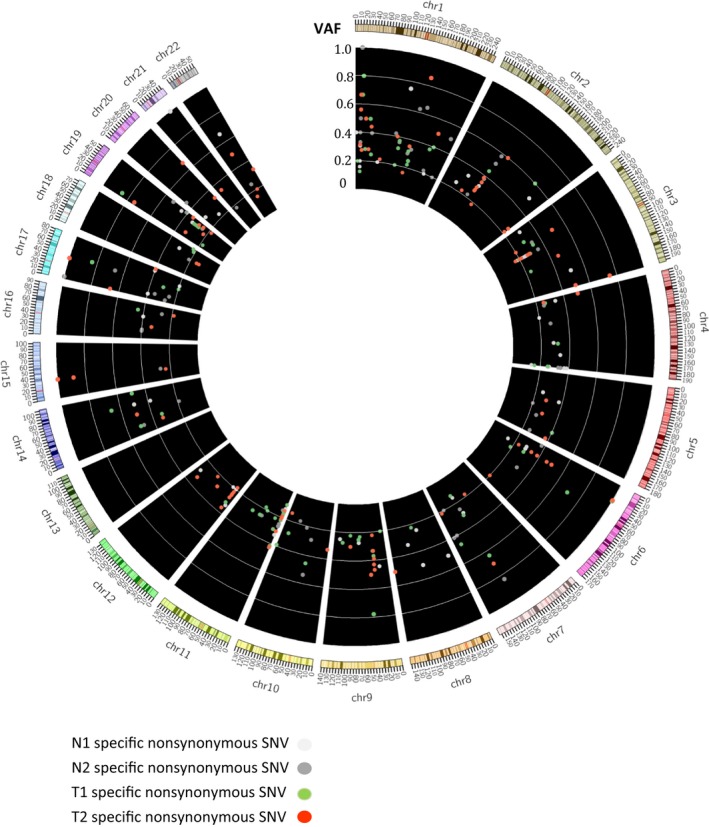
The genomic variants of each sample. There are five circles, representing each sample

In this study, we collect one blood sample and four frozen tissue samples from the 52‐year‐old female never smoking hereditary lung adenocarcinoma patient receiving operation twice. We defined 1665 mutations of all samples after 1000 genomes filtering. Without any known actionable mutations were detected in the five samples, such as *EGFR*, *KRAS*, *BRAF*, *HER2,* and *EML4‐ALK* in the tumor tissues. The next generation sequencer technology was applied to do whole genome sequence for the blood DNA, first cancer tissue (T1) and adjacent normal tissue (N1), relapse cancer tissue (T2), and adjacent normal tissue (N2) of this patient, respectively. The average coverage depth of five samples is ~25X and number of detected mutations is around 3 million. They were mapped to the reference genome (NCBI build 37, HG19) at an over 80% mapping rate for confident variant calling (Supplementary Table S1). Figure 1 showed the flowchart of tumor mutational burden (TMB) identification in this study. TMB was defined as the number of somatic mutation in coding region. Synonymous mutations could not be directly involved in creating neoantigens, and we focused on nonsynonymous mutation. Our approach for variants calling was intersection of the two vcf files (GATK + samtools).The somatic variants calling were filtering based on 1000G and our blood sample (germline mutation) to identify variants. Since our study was biased toward genes with functional mutations in cancer, we separated the variants on the coding regions (exons) for the step selection. The exonic alterations of each sample were presented as Circos plots in Figure 2. Previous studies indicated that the nonsilent mutations derived in regulatory regions were believed to cause the phenotypic differences.^21^ The Supplementary Table S2 also showed the number of the exonic mutations in each sample. After comparing with germline background (blood DNA), and normal part tissues, the 215 tumor specific functional mutations (missense and nonsense mutations) were identified. TMB calculation was based on the tumor specific functional mutations in the TCGA cohort.

### 3.2 Single‐nucleotide substitution pattern

**Figure 3:**
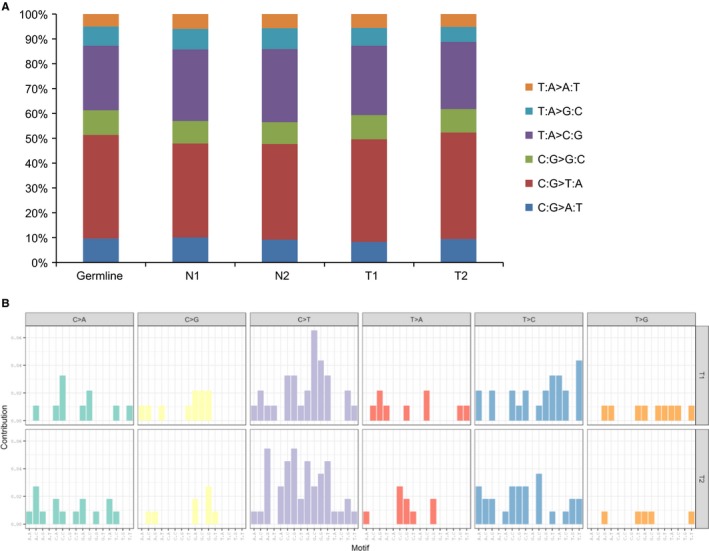
Somatic mutation pattern (A) in each sample and mutation signatures in tumor tissues (B) by WGS

**Figure 4:**
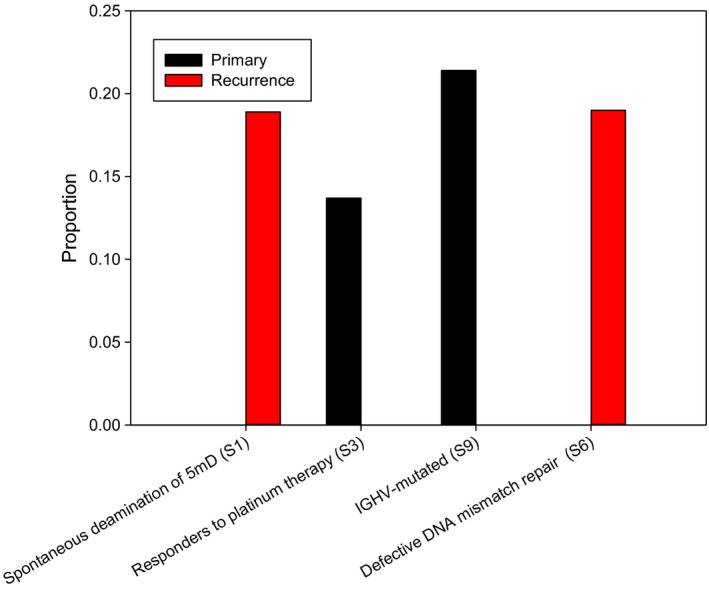
The top three COSMIC cancer mutation signature in primary (T1) and recurrent (T2) tissues

Previous study showed that C:G>A:T transversions in the lung cancer tissues from never smokers is significantly lower than that of smokers.^4^ We also found that C:G>T:A (40%) and T:A>C:G (28%) base substitutions were predominated in the each sample. Thus, the relatively higher frequency of C:G>T:A and T:A>C:G transitions compared with C:G>A:T transversions in the nonsmoking patient. The single nucleotide substitution pattern was similar in each sample (Figure 3A). We used deconstructSigs to identify of mutational signatures.^22^ Correlation with the 30 curated mutation signatures according to Somatic Mutations in Cancer (COSMIC) database^23^ indicated that a primarily C to T transition signature (Figure 3B). The tumor mutation signature spectrums were different composition in primary and recurrent tumors (Supplementary Figure S1). Three dominate signatures in primary tumor (T1) were signature 9, 3, and 15 and in recurrence tumor (T2) were signature 1, 5, and 6. The signature 9 was associated with somatic immunoglobulin gene hypermutation and other signatures were involved in defective DNA repaired process.^22^ Signature 3 exhibited in responders to platinum therapy and the patient had response with neoadjuvant chemotherapy (cisplatin) before resection 24, 25(Figure 4). We indicated signature 6 associated with defective DNA mismatch repair in recurrent tumor tissues. Because of the patient received chemotherapy after surgery, the higher ratio of defective DNA repair signatures in the recurrent tissues could be attributed to the therapy.

### 3.3 Functional enrichment analysis of somatic SNVs

We used the IPA (Qiagen, Redwood City, CA), a web‐based computational platform designed for system biology, to conduct biological function enrichment analysis of exonic somatic SNVs. We input the set of 846 genes (1665 variants) and used the IPA with the default settings. The results show that somatic mutated genes were enriched in antigen presentation pathway (Table 1). Table 1 list the top five significantly enriched pathways (*P* < 0.001). The results indicate the Antigen Presentation Pathways, (*P* = 9.08E‐08, input/total nodes = 11/38), which has a much higher ratio of root to total nodes and significant P‐value. The antigen presentation pathway leading to the association of the MHC (major histocompatibility complex) molecule differs for class I and class II MHC. HLA is the major histocompatibility complex (MHC) specific to humans. Antigen presentation pathway played an important role in the immune system involves the processing of antigen, association of processed antigen with MHC molecules and cell surface presentation of the antigen in the context of MHC to T cells. Moreover, we determined the sequencing coverage of HLA regions were nearly 95% (Supplementary Table S3). This two paired normal tumor comparison could identify tumorigenesis and recurrent mutations when compared with germline and normal part mutation profiles. The most significant pathway of enrichment analysis using T1 specific somatic variants is protein kinase A (*PKA*) signaling (*P* = 7.39E‐03). There were four gene (*CDC27*, *FLNB*,* PTPRC,* and *PTPRD*) involved the pathway. The *PKA*signaling is the serine‐threonine protein kinase family, and is involved in the control of a variety of cellular processes. The pathway has been implicated in the initiation and progression of tumors,^26^, ^27^, ^28^ and *PKA*derived mammary tumorigenesis through *Src* activation.^29^ Additionally, *Src*activation enhanced YAP1 expression^30^ and modulated the initial EGFR TKI response in lung cancer.^31^ This has been suggested that T1 specific variants associated with tumorigenesis pathway. T2 specific variants were enriched in the cancer metastasis signaling (*P* = 4.33E‐03) including the matrix metalloproteases 11 (*MMP11*), low density lipoprotein receptor‐related protein 1(*LRP1*), mutS homolog 3 (*MSH3*), and son of sevenless homolog 2 (*SOS2*). This result showed that the tumor recurrence might be related with tumor metastasis mechanism (Supplementary Table S4).

**Table 1:** Top five pathway of enrichment analysis by functional ontology enrichment tool in ingenuity pathway analysis

Pathway name	*P*	Ratio
Antigen presentation pathway	9.08E‐08	11/38
Crosstalk between dendritic cells and natural killer cells	1.18E‐04	12/89
Calcium‐induced T lymphocyte apoptosis	1.62E‐04	10/66
Th1 pathway	1.66E‐04	15/153
B cell development	2.17E‐04	7/35

### 3.4 HLA profiles and phylogenetic applications of HLA‐A

**Figure 5:**
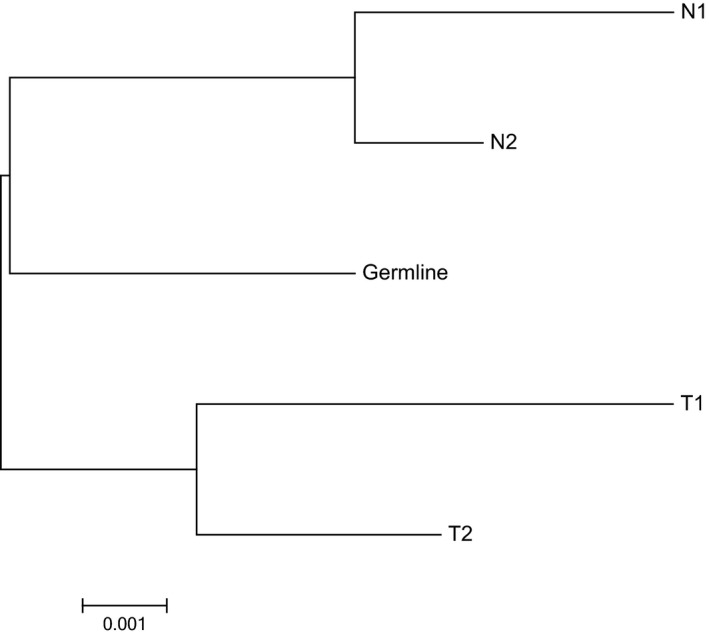
Maximum‐likelihood phylogenetic relationships of samples using HLA regions

The HLA region located on chromosome 6p21.31 with the class I and II region. HLA class I (A, B, and C) and class II (DRB1, DQB1, and DPB1) antigens are involved in the immune response. The studies of cancer immunology have moved forward to the identification of numerous tumor‐associated antigens.^32^, ^33^ The function of the HLA complex involved the immune response such as the immune response to environmental pathogens and in autoimmune disease. HLA was originally identified in the organ transplant rejection.^34^ HLA has been reported in the etiology of diseases, including immune diseases,^35^ cancers,^36^, ^37^ and infectious diseases.^38^, ^39^ Phylogenetic analysis was based on the HLA sequencing data of the five samples. The maximum likelihood approach was used to determine the phylogenetic relationship among different samples. The phylogenetic tree generated the differences between HLA‐A allele frequencies in different samples (Figure 5). The edge lengths displayed in the phylogram indicated that the amount of change occurred along each branch. Overall, the phylogenetic tree exhibits a clearer picture showing distinct normal and tumor parts.

### 3.5 Tumor mutation load

**Figure 6:**
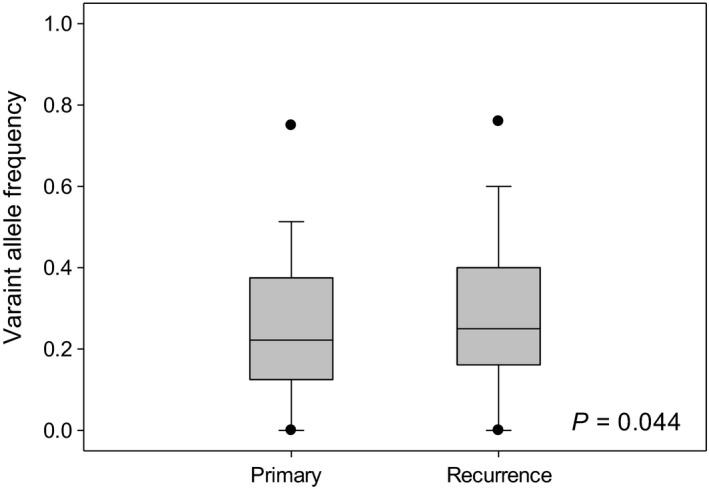
Allele frequency of 215 nonsilent somatic variants in the primary and recurrence lung adenocarcinoma tissues

**Figure 7:**
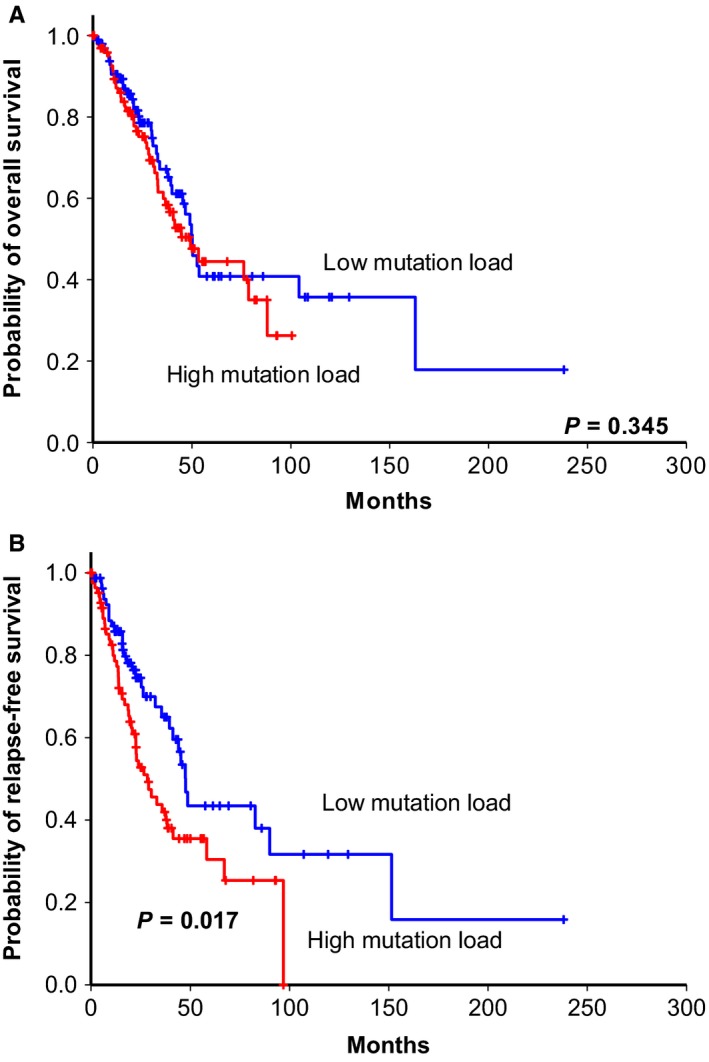
Kaplan‐Meier overall survival (A) and disease‐free (B) curves of tumor specific mutation load in EGFR wild type lung cancer cohorts from TCGA

We identified the 215 tumor specific mutations in primary (T1) and recurrent (T2) tumor tissues. They were harbored by 162 genes. The sum of allele frequency in tumor specific somatic mutations was significantly higher in the recurrence (T2) part than primary (T1) part (*P* = 0.04, paired *t* test, Figure 6). Then using TCGA‐LUAD data, we identified the mutations in these genes (Supplementary Figure S2). Furthermore, we demonstrated that patients with high allele frequency of missense and nonsense mutations in tumor specific parts had a significantly shorter relapse‐free survival time than the low mutation frequency group for the *EGFR* wild‐type lung cancer patients (*P* = 0.017, Figure 7), though no significant difference present in overall survival (*P* = 0.67, Figure 7). In contrast, for the *EGFR* mutation patients, the mutation load difference did not correlate with patient overall and relapse‐free survival (Supplementary Figure S3). We conducted a multivariate Cox regression analysis to search for independent prognostic factors associated with survival. The result showed that patients predicted to have higher mutation load had a significantly increased risk for poor survival. The adjusted hazard ratio (HR) is 1.86 (*P* = 0.01) for the mutation load signature (Table 2). Portraying the spectrum of tumor recurrence mutations in the same patient without the oncogenic lung cancer driver mutations such as EGFR, KRAS, and EML4‐ALK is an integrative step of understanding lung cancer tumor progression. But the literature is lacking. Aiming to fill up the gap, we demonstrated that high tumor mutation load might mediate tumor progression and recurrence by activating the immune escape mechanism and that high mutation load in the EGFR wild‐type patients correlated with worse relapse‐free survival.

**Table 2:** Multivariate Cox regression analysis of the mutation load signature for predicting relapse‐free survival in lung cancer patients

Variable	Hazard ratio	95% CI	*P*
Lung cancer (n = 71)			
Mutation load (Low vs High)	1.86	3.03‐1.13	**0.01**
Age (≦60 yr vs > 60 yr)	1.33	2.19‐0.81	0.26
Gender (Male vs Female)	1.33	0.48‐3.68	0.34
Stage (1,2 vs 3,4)	1.17	0.64‐2.13	0.62

Bold indicates significant results (*P* < 0.05).

## 4 DISCUSSION

In this study we compared blood, normal‐tumor pairs with primary and relapse tumors from a hereditary lung adenocarcinoma patient, aiming to characterize the similarities and differences between these tissues. No mutations in *EGFR*, *KRAS*, *HER2*, *BRAF,* and *EML4‐ALK* were found by mass spectrometry, Ventana method and whole genome sequence. Interestingly, the whole genome sequence analysis indicated that this patient did not carry any known oncogenetic driver mutations beside germline Yap1 mutation in tumor tissues (Supplementary Table S2). Thus the tumor recurrence and progression in this patient might not be through the known oncogenetic pathway.

The concept of mutational burden was examined in our study. Previous investigations have explored the potential immunogenicity of tumor mutations.^40^, ^41^ Antigen frequency of HLA class I alleles correlated with the prognosis and pathological factors in lung cancer patients. Incidence of HLA Class I and Class II alleles indicated tumor spreading to other organs or to lymph nodes in lung cancer patients.^42^ HLA Class I alleles may affect postoperative prognosis.^43^ Our results supported the hypothesis that HLA alleles play a role in lung cancer relapse.

The findings of mutation burden enriched in Antigen Presentation Pathways by our study may offer new biological insight to cancer relapse and immunotherapy targets. The process of tumorigenesis depends both on the genetic alterations of tumors and the interaction with their immunological environment. HLA genes play an important role in tumor's immune escape.^44^, ^45^ One meta‐analysis demonstrated that the immune checkpoint inhibitors significantly prolonged survival in the *EGFR* wild‐type subgroup but not in the *EGFR*‐mutant subgroup.^46^ For the TCGA‐LUAD data the mean mutation load is 0.54 in *EGFR*mutation group (n = 24), and 0.76 in *EGFR*wild type group (N = 187) (*P* = 0.516). There may be a limitation to evaluating *EGFR*mutation from Caucasian specimens, but the patients with high recurrent tumor‐specific mutation burden had worse relapse‐free survival. To our knowledge, it is the first study to describe the recurrent tumor‐specific mutation burden form the same patient tissue in hereditary lung cancer by whole genome sequencing. Limited by the genetic material availability, we only evaluated 1 patient with different part tissues. We demonstrated that patients with high mutation burdens in the genes with functional enrichment in immune response inversely correlated with relapse‐free survival. Further investigation on the molecular mechanisms behind how tumor cells escape from immune surveillance will provide important insight for designing immunotherapeutic strategies. Moreover, analysis of tumor mutational burden may be important for investigating not only etiology, but also therapy and prognosis. The mutation might inhibit immune recognition of this epitope by the well‐characterized escape mechanism involving impairment of peptide‐TCR‐HLA interactions.

## CONFLICT OF INTEREST

All authors declared no conflicts of interest.
