# Supplementary material for: Tumor mutation burden and recurrent tumors in hereditary lung cancer
Source: Cancer Med. 2019 Apr 2;8(5):2179–87. doi: 10.1002/cam4.2120 (PMC6536970; doi:10.1002/cam4.2120)
Supplement: Supplementary file 2 [file CAM4-8-2179-s002.docx]

Figure S1. The COSMIC cancer mutation signature in primary (T1) and recurrent (T2) tissues.

Figure S2. Mutation loads from TCGA LUAD database.

Figure S3. Kaplan–Meier overall survival (A) and disease-free (B) curves of tumor specific mutation load in EGFR mutation lung cancer cohorts from TCGA.
